# Case Report: Neurobrucellosis Presenting as Malignancy

**DOI:** 10.4269/ajtmh.23-0684

**Published:** 2024-06-11

**Authors:** Andrea S. Salcedo, Xosse Carreras, Takaaki Kobayashi, Jorge L. Salinas, Sara Muñoz, Nelson Diaz, Jorge Alave

**Affiliations:** ^1^ Universidad Peruana de Ciencias Aplicadas, Lima, Peru;; ^2^ Department of Internal Medicine, University of Iowa Hospitals and Clinics, Iowa City, Iowa;; ^3^ Stanford University, Stanford, California;; ^4^ Clinica Good Hope, Lima, Peru;; ^5^ Universidad Peruana Union, Lima, Peru and Clinica Good Hope, Lima, Peru;; ^6^ Universidad Peruana Union, Lima, Peru

## Abstract

Neurobrucellosis, caused by *Brucella* species, is a zoonotic infection that may involve the central nervous system. Although uncommon, it can manifest as a solitary intracranial mass. We report a case of neurobrucellosis in a 25-year-old woman from Peru who presented with headache, weight loss, and right-side hemiparesis and paresthesia. A contrast-enhanced magnetic resonance imaging scan revealed an intracerebral mass in the left temporal lobe. Serum testing subsequently were positive. Brain biopsy demonstrated non-necrotizing granulomas without malignant cells. Neurobrucellosis should be considered in the differential diagnosis of brain space occupying lesions in endemic countries.

## INTRODUCTION

Brucellosis involving the central nervous system (CNS) is known as neurobrucellosis.^1^ The frequency of neurobrucellosis ranges from 0.5% and 25% for all types of brucellosis.^2^ Commonly reported presentation for neuroburucellosis include meningoencephalitis, radiculitis, and cranial neuropathies.^2^ In rare presentations, neurobrucellosis can present as a solitary intracranial mass. We report a case of neurobrucellosis in an immunocompetent patient who presented intracranial hypertension syndrome due to a space-occupying lesion in the brain.

## CASE REPORT

A 25-year-old, previously healthy woman from Lima, Peru, presented to the emergency department with headache, weight loss, and right-side paresthesia and hemiparesis. One month before admission, she developed a global headache of gradual onset and moderate intensity, present through most of the day. Three days before admission, the headache significantly worsened and was associated with nausea and vomiting that interrupted her sleep. She also developed weakness of the right side of her body and an abnormal sensation on the left side of her face and neck. She reported having lost approximately 20 kg over the previous 3 months. She did not have any other past medical or surgical history and was not taking any medications except for ibuprofen that she had started recently for headache. She denied any sick contacts, recent travel, or recreational drug use. She lived at home with a dog and a cat and worked as an office employee. Her diet included dairy products and different types of cheese, possibly including nonpasteurized varieties.

On physical examination, her temperature was 37.1°C (98.7°F), and her heart rate was 70 beats per minute. She appeared somnolent, and abdominal examination revealed hepatomegaly. Neurological examination revealed photophobia, mild hemiparesis, and paresthesia in the right arm and leg. Pupils and cranial nerves were normal. Babinski’s sign and clonus were not observed. Bilateral osteotendinous reflexes of the upper and lower extremities were normal.

**Figure 1.**
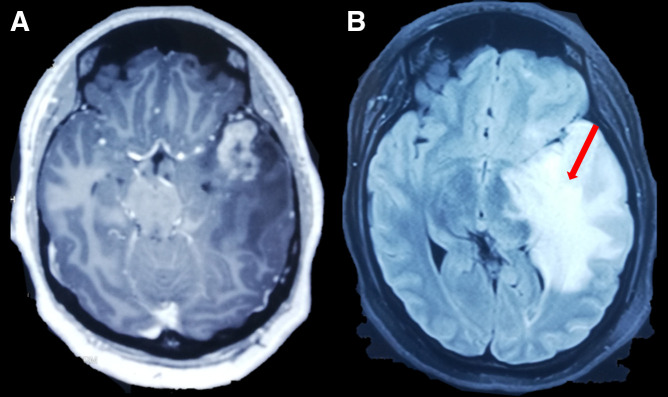
(**A**) Axial T1-weighted contrast-enhanced magnetic resonance imaging reported cerebral mass in left temporal lobe with peripheral edema (red arrow). (**B**) Fluid attenuated inversion recovery sequence showing expansive lesion in temporal lobe (red arrow). Both sequences showed a shift of midline.

**Figure 2.**
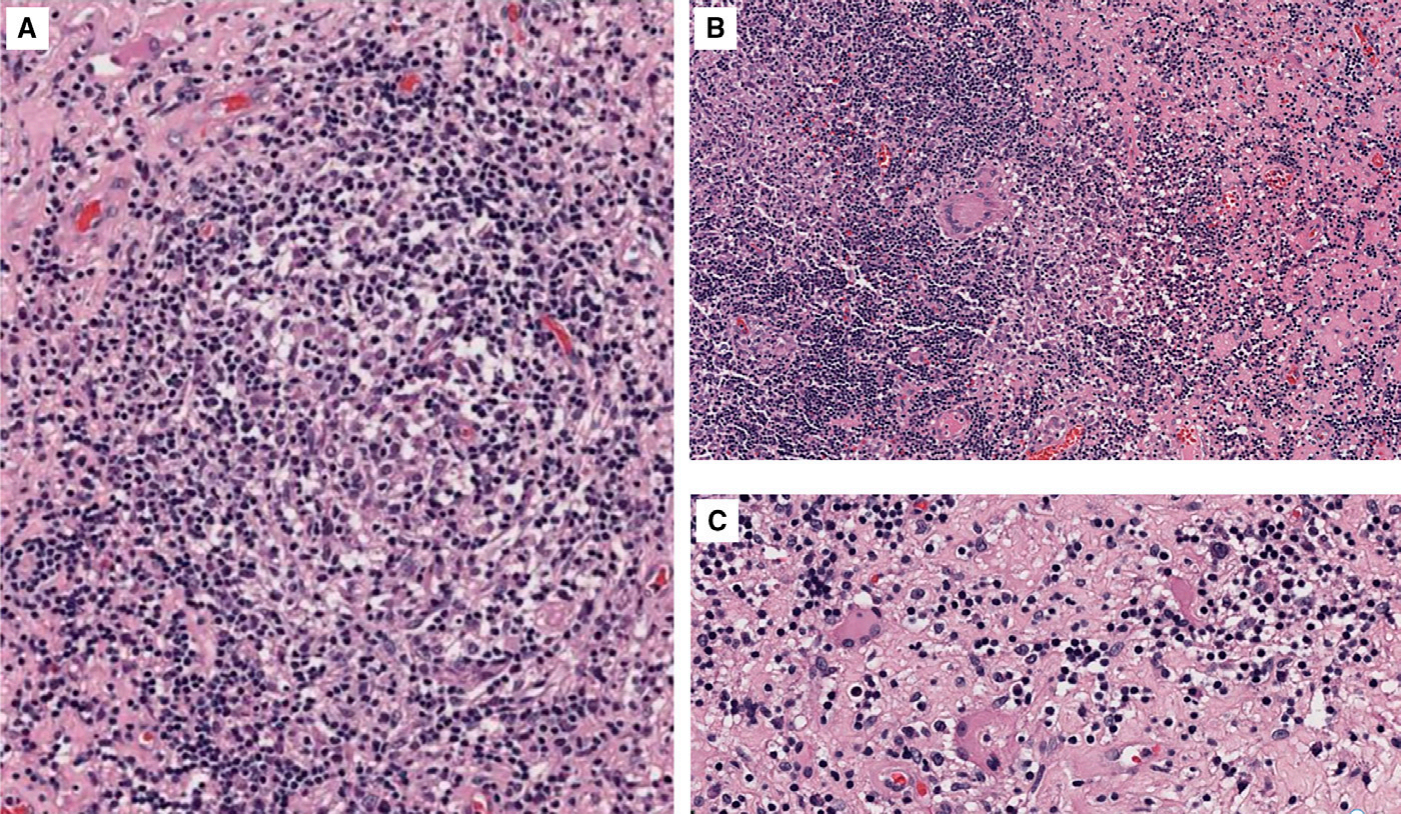
(**A–C**) Hematoxylin and eosin–stained microphotography of brain parenchyma revealed a dense lymphocytic inflammatory infiltrate with the formation of non-necrotizing granulomas and multinucleated giant cells. Congestion and edema of the neuropil were also noticed.

**Figure 3.**
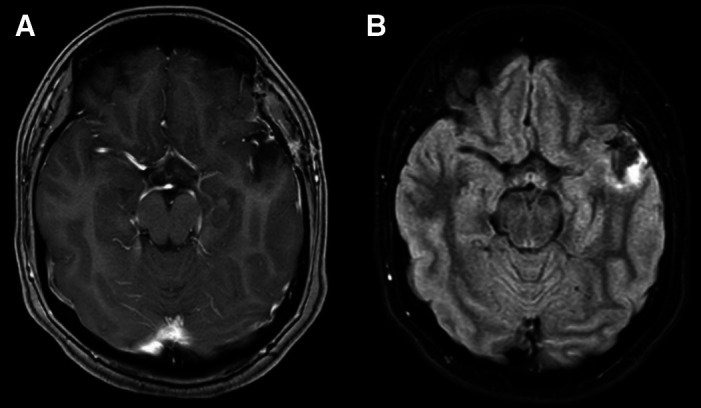
At 6-month follow-up, (**A**) axial T1-weighted contrast-enhanced magnetic resonance imaging and (**B**) fluid attenuated inversion recovery sequence showed a noticeable decrease in the lesion of the temporal lobe.

On admission, basic laboratory tests showed leukopenia 1.3 × 10^3^ cells/µL (reference value: 4–10 × 10^3^ cells/*µ*L) but no other hematologic or basic metabolic abnormalities. Contrast-enhanced head magnetic resonance imaging (MRI) revealed an intracerebral mass in the left temporal lobe with marked regional vasogenic edema, midbrain compression, and ventricular collapse (Figure 1). A primary or secondary brain tumor was suspected, and mannitol solution was started due to intracranial hypertension syndrome. Because of the clinical presentation and imaging findings, serology tests were performed to rule out infectious causes. *Brucella abortus* IgM and IgG ELISA results were 3.1 U/mL (reference value: <2 U/mL) and 2.9 U/mL (reference value: <2 U/mL), respectively. Bengal rose, *Brucella* latex agglutination test and tube agglutinations were also positive at 1/160 (NV: <1/40) and 1/80 (NV: <1/40), respectively, whereas serum *Brucella* polymerase chain reaction (PCR) was negative. Other infectious diseases tests were negative, including blood cultures, *Toxoplasma* sp. IgM and IgG, *Cysticercosis* ELISA, HIV ELISA, human T-lymphotropic virus ELISA, and interferon-gamma release assay for *Mycobacterium tuberculosis*. Brucellosis was suspected, and she was started on empiric antibiotic treatment with doxycycline and gentamicin. The patient underwent a brain biopsy of the left temporal lobe. During the procedure, a grayish vascularized fibrous lesion with surrounding edema was observed. The histopathological examination showed cerebral parenchyma with dense lymphocytic infiltrate and a granulomatous pattern (Figure 2). No malignant cells were seen and Ziehl–Neelsen stain, periodic acid-Schiff stain, Grocott’s methenamine silver stain and *M. tuberculosis* PCR using artus *M. tuberculosis* RG PCR kit (Qiagen, Germany) were all negative. No PCR for *Brucella* spp. was performed in the resected tissue. Bacterial, fungal, and acid-fast bacillus culture from the biopsy samples remained negative. Because of the positive serology for brucellosis and negative work-up for other infectious etiology, she was diagnosed with neurobrucellosis and had the majority of the space-occupying lesion resected by neurosurgeons. Antibiotic treatment was changed to triple therapy with ceftriaxone, doxycycline, and cotrimoxazole. After 2 weeks, ceftriaxone was discontinued, and doxycycline and cotrimoxazole were continued for a total of 6 months, resulting in significant clinical improvement. After 6 months, a repeat head MRI was performed, which showed a decrease in the lesion of the temporal lobe (Figure 3). At 2-year follow-up, she was symptom free without weakness or abnormal sensation, and there has been no recurrence of disease.

## DISCUSSION

Brucellosis is a zoonotic infection mainly acquired through the consumption of food products such as unpasteurized milk or direct contact with tissue or fluid of infected animals.^3^ It is known to be endemic to North Africa, the Mediterranean, and South America.^1^ Peru reported 3,985 cases between 2009 and 2018.^4^ The incubation period is usually between 2 and 4 weeks, but ranges from 5 days to 6 months.^5^

Brucellosis has multisystemic complications compromising any organ, such as the spine (spondylodiscitis), heart (endocarditis), testicles (epididymorchitis), lungs (pneumonia), and rarely the CNS. In one case series, fever was reported in 76% of brucellosis patients.^6^ In another case series involving neurobrucellosis patients, fever was reported in 79% of patients.^2^ *Brucella* sp. shows a predilection for the meninges,^7^ and neurobrucellosis usually presents as meningitis or meningoencephalitis (up to 3–5%).^3^ However, it rarely appears as a brain mass. Symptoms are diverse including headache, back pain, areflexia, myelitis, and cranial nerve involvement, among others.

The diagnosis of neurobrucellosis can be made by blood culture, cerebrospinal fluid culture (CSF), serological agglutination tests (SAT), 2-mercapto-ethanol test, Bengal rose, and/or ELISA. However, while blood and CSF cultures are considered the gold standard, the positivity of these tests is only up to 20%.^7^ Therefore, diagnosis is usually established by the detection of *Brucella* antibodies by agglutination test (Wright or Coombs agglutination with a minimum titer of 1:160) as in our case. This test presents a low sensitivity but a high specificity (100%).^8^ Other serological tests such as ELISA, positive in this case, aids in the diagnosis yielding a sensitivity and specificity up to ∼100% for neurobrucellosis.^8^ Brain biopsy is important to exclude other diagnoses, and typical histopathology finding is a noncaseating granuloma.^9^ A presumptive diagnosis is usually made by clinical examination and serological tests to avoid invasive procedures. There are different diagnostic criteria for brucellosis. For example, the US CDC established that a confirmed case requires a positive culture or a 4-fold increase in antibody production 2 weeks apart.^5^ However, the China CDC considers a case confirmed when a probable case has an SAT ≥100, Coombs IgG, or a positive culture.^10^

Neurobrucellosis presenting as a cerebral mass is rare. A PubMed and Google search using the terms “neurobrucellosis” and “mass” or “tumor” in both English and Spanish yielded seven case reports, in addition to ours, in which two were pediatric cases (Table 1). Half of patients were female (four of eight). Mean age was 24 years (SD: 12.7; range: 14–52), and interestingly, four of the eight patients were from Peru. The most common neurological manifestations were headache, hemiparesis, seizures, and/or signs of intracranial hypertension.^7^^–^^9^^,^^11^^,^^12^ Rarely deterioration in visual function was found. The diagnosis of neurobrucellosis, as in our case, was made by complementing serological tests in half of the reported cases, and only three cases were diagnosed by culture,^7^^,^^8^ of which two were tissue cultures and one was isolated from blood. In five cases, complete excision of the mass was performed, and the pathology showed brain parenchyma with lymphocytic infiltrate and nonspecific granulomatous reactions in all five cases.^7^^,^^8^^,^^11^^,^^12^ No mortality was reported.

**Table 1:** Summary of Eight Neurobrucellosis Cases Presenting as a Cerebral Mass

Author Age/Sex Country	Clinical Manifestations and Neurologic Signs	Diagnosis	Pathology Results	Treatment	Outcome
Algahtani et al.^9^ 52/F, Saudi Arabia	Headache, dizziness, partial seizures/mild papilledema	Serology IgM positive (12.2 U/mL), IgG positive (127.4 U/mL), and CSF *Brucella* antigens positive (1:40); *Brucella* antibodies, oligoclonal bands positive, five well-defined gamma restriction bands for *Brucella*	Not performed	Rifampicin (600 mg/day) plus doxycycline (100 mg twice a day) and TMP-SMX (960 mg twice a day) for 6 months	Favorable
Miguel et al.^11^ 39/M, Peru	Headache, weakness in right upper and lower body, Broca’s aphasia, right hemiparesis, diminished tendon reflexes in right lower limb	Serology IgM in CSF 1.38 and serum 2.12, and IgG in serum 3.05	Granulomatous encephalitis	Tumor resection and doxycycline (200 mg/day) plus rifampin (600 mg/day) and gentamycin (240 mg/day) for 2 months	Favorable
Erdem et al.^7^ 20/F, Turkey	Headache, left arm and leg numbness/left hemiparesis	Blood culture: *Brucella melitensis*, CFS Wright agglutination test positive (1:320)	Non-granulomatous encephalitis: diffuse lymphocytic infiltrates and perivascular lymphocytic cuffing	Rifampin (600 mg/day) plus TMP–SMX (640–3,200 mg/day) and ceftriaxone (2 g/day) for 3 weeks; thereafter, ceftriaxone was changed for doxycycline (200 mg/day) for 6 months	Favorable
Martı´nez-Chamorro et al.^12^ 14/M, Spain	Tonic–clonic seizures/mild hypoesthesia of the left arm	Serology agglutination positive (1:2560), *Brucella* CSF titer positive (1:20)	Chronic non-granulomatous inflammatory changes	Rifampin (600 mg/day) and oral TMP–SMX (640–3,200 mg/day) for 6 weeks	Favorable
Sohn et al.^8^ 26/M, Peru	Headache, periorbital pain/generalized tonic–clonic seizures	Tissue culture *Brucella* spp., tissue PCR *Brucella* spp., serology positive agglutination test (1:160)	Granulomas with multinucleated giant cells	Tetracycline and rifampin for 2 months	Favorable
Sohn et al.^8^ 15/M, Peru	Headaches, vomiting, deterioration in visual function/right homonymous hemianopsia, optic nerve atrophy, and major visual impairment	Tissue PCR: *Brucella* spp., serology negative	Granulomas with multinucleated giant cells	Treatment with rifampin, doxycycline, and intravenous gentamicin for 1 week; gentamicin was changed for TMP-SMX and continued for 1 year	Favorable
Çiftçi et al.^13^ 25/F, Turkey	Bilateral hearing loss, headache, diplopia, gradual loss of vision, nystagmus, generalized weakness, amenorrhea and galactorrhea	*Brucella* agglutination positive (>1: 320), *Brucella* serology CSF positive (1:160), IgG oligoclonal band positive in blood and CSF	Not performed	Tetracycline and TMP-SMX for 10 weeks	Favorable
Present case, 25/F, Peru	Headache, hyporexia, weakness and paresthesia in right hemibody, left hemiface and neck, weight loss, photophobia, right hemiparesis	Serology IgM positive (3.1 U/mL) and IgG positive (2.9 U/mL, Bengal rose positive, *Brucella* latex agglutination test positive (1/160) and tube agglutinations positive (1/40)	Lymphocytic infiltrate and unspecific granulomatous reaction	Ceftriaxone, for 2 weeks, with doxycycline plus TMP-SMX for 6 months	Favorable

CSF = cerebral spinal fluid; F = female; M = male; TMP-SMX = trimethoprim–sulfamethoxazole.

The optimal approach to treatment of neurobrucellosis is uncertain; data are limited to retrospective and observational studies. In the specific case of brain space-occupying lesions due to neurobrucelosis, different combinations and durations of treatment have been used, including surgical resection. In the preceding case review, treatment was varied including the combination of rifampin, doxycycline, trimethoprim–sulfamethoxazole (TMP-SMX), gentamicin, and ceftriaxone. Triple therapy was widely used. All cases used rifampin in the treatment except for our case and that of Çiftçi et al.,^13^ because new evidence has shown that the use of TMP-SMX plus doxycycline have similar failure and relapse rates.^14^ Only one case, besides ours, used ceftriaxone-based triple therapy. Erdam et al.^15^ found that in patients with neurobrucellosis, the use of ceftriaxone-based regimens had lower rates of failure and recurrences. Likewise, Fatani et al.^3^ concluded that the use of ceftriaxone added to the basal regimen in complicated brucellosis such as neurobrucellosis is a reasonable option. On the other hand, treatment duration was different across the case reports, lasting from 6 weeks up to 1 year. According to Zhao et al.,^16^ the minimum duration of treatment is 6 weeks and depends on the patient’s response and normalization of CSF. Finally, surgical resection of the mass is controversial because there is no literature advocating for surgical resection. However, in this review, we found five cases that performed it with favorable outcomes in all patients.

In conclusion, neurobrucellosis should be considered in the differential diagnosis of brain space-occupying lesions in endemic countries. Serology tests and CSF analysis for brucellosis remain useful and sensitive diagnostic tools. Although the data are limited, triple therapy has shown to be superior to dual regimens for successful treatment. Despite its rare presentation, the outcome of this disease appears to be favorable.
